# Therapeutic Effects of Gingival Mesenchymal Stem Cells and Their Exosomes in a Chimeric Model of Rheumatoid Arthritis

**DOI:** 10.21203/rs.3.rs-3121787/v1

**Published:** 2023-07-05

**Authors:** Shane Bruckner, Vittoria M Capria, Braden Zeno, Binnaz Leblebicioglu, Kanu Goyal, William K Vasileff, Hisham Awan, William L Willis, Latha P Ganesan, Wael N Jarjour

**Affiliations:** The Ohio State University Wexner Medical Center; The Ohio State University; The Ohio State University Wexner Medical Center; The Ohio State University; The Ohio State University Wexner Medical Center; The Ohio State University Wexner Medical Center; The Ohio State University Wexner Medical Center; The Ohio State University Wexner Medical Center; The Ohio State University Wexner Medical Center; The Ohio State University Wexner Medical Center

**Keywords:** Rheumatoid Arthritis, Synovial fibroblasts, Gingival-Derived Mesenchymal Stem Cells, Exosomes

## Abstract

**Background:**

Rheumatoid arthritis is a chronic systemic autoimmune disease that involves transformation of the lining of synovial joints into an invasive and destructive tissue. Synovial fibroblasts become transformed, invading and destroying bone and cartilage of the affected joint(s). Due to the significant role these cells play in the progression of the disease process, developing a therapeutic strategy to target and inhibit their invasive destructive nature could help patients who are affiicted with this debilitating disease. Gingival-derived mesenchymal stem cells are known to possess immunomodulatory properties and have been studied extensively as potential cell-based therapeutics for several autoimmune disorders.

**Methods:**

A chimeric human/mouse model of synovitis was created by surgically implanting SCID mice with a piece of human articular cartilage surrounded by RASF. Mice were injected once with either GMSC or GMSCExo at 5–7 days post-implantation. Histology and IHC were used to assess RASF invasion of the cartilage. Flow cytometry was used to understand the homing ability of GMSC in vivo and the incidence of apoptosis of RASF in vitro.

**Results:**

We demonstrate that both GMSC and GMSCExo are potent inhibitors of the deleterious effects of RASF. Both treatments were effective in inhibiting the invasive destructive properties of RASF as well as the potential of these cells to migrate to secondary locations and attack the cartilage. GMSC home to the site of the implant and induce programmed cell death of the RASF.

**Conclusions:**

Our results indicate that both GMSC and GMSCExo can block the pathological effects of RASF in this chimeric model of RA. A single dose of either GMSC or GMSCExo can inhibit the deleterious effects of RASF. These treatments can also block the invasive migration of the RASF, suggesting that they can inhibit the spread of RA to other joints. Because the gingival tissue is harvested with little difficulty, relatively small amounts of tissue are required to expand the cells, the simple in vitro expansion process, and the increasing technological advances in the production of therapeutic exosomes, we believe that GMSCExo are excellent candidates as a potential therapeutic for RA.

## Background

Rheumatoid arthritis (RA) is a chronic inflammatory autoimmune disease typically involving multiple joints in which the synovium becomes inflamed and hyperplastic (1). This destructive invasive synovial pannus irreversibly destroys the articular cartilage and marginal bone, leading to significant disability if under-treated (1–4). The availability of a variety of novel therapies combined with early recognition of the disease facilitates remission for many patients, however there are still those patients who do not respond with such success or who develop severe side effects or a relapse of their chronic condition despite optimal initial control of the disease (5). It is therefore of utmost importance to continue the search for therapeutic interventions that can cure RA or, at a minimum, have a higher prevalence of long-lasting remission for most patients (6, 7).

There is a large body of evidence that suggests a crucial role for activated synovial fibroblasts (RASF) in mediating both direct tissue injury and perpetuation of the complex disease process in RA (1, 8–12). While it is well established that many cell types are involved in the pathogenesis of RA (13–16), RASF isolated from RA patients and maintained *in vitro* attack the articular cartilage and deeply invade and degrade the cartilage matrix independently of other cell types or inflammatory factors that are present in *vivo in* the diseased tissue (17–20). RASF have also been shown to migrate to other joint locations *in vivo* and invade and degrade cartilage similarly to the primary site (21). This evidence implicates RASF as a primary driver of joint destruction and the polyarticular nature of the disease, therefore RASF are a potentially impactful target for the treatment of RA.

Human gingival-derived mesenchymal stem cells (GMSC) are promising therapeutic cell treatments for autoimmune diseases due to their immunomodulatory capacity (22, 23). These cells are similar in their immunomodulatory potential to their well-studied bone marrow-derived and adipose-derived mesenchymal stem cell counterparts but do not possess many of the negative features associated with these other stem cell types as well as possessing several advantageous characteristics (24–26). In addition, the natural inflammatory microenvironment from which GMSC are derived, potentially confers stronger immunoregulatory capabilities as compared to these other mesenchymal stem cell types (27). Others have demonstrated the ability of GMSC, and exosomes derived from GMSCs (GMSCExo) to suppress the deleterious *in vivo* effects of the collagen induced arthritis model in mice (27, 28). The aim of this study was to test whether the destructive invasive effects of RASF in an *in vivo* chimeric humanized mouse model of RA could be modulated by treating with either GMSC or GMSCExo derived from healthy human subjects. In addition, we analyzed the effects of both GMSC and GMSCExo on the ability of RASF to migrate to secondary locations.

Our results indicate that both GMSC and GMSCExo are potent inhibitors of the deleterious effects of RASF. More specifically, a single dose of either treatment inhibited the RASF from invading the cartilage at the primary implant site and inhibited the invasive migration of the RASF to the secondary cartilage implant. We also present evidence of a mechanism of action of these treatments via induction of RASF cell death. As clinical treatments for RA, exosomes derived from GMSC would be free of potential complications associated with cellular therapies and would not only have the potential to inhibit the progression of existing joint destruction by RASF, but also stop them from spreading the disease to other secondary joint locations.

## Methods

### GMSC Isolation:

Human gingival tissue samples were obtained as remnants of clinically healthy discarded gingiva following routine periodontal gingivectomy procedures at the College of Dentistry, The Ohio State University under an approved Institutional Review Board (IRB) protocol. Gingiva-derived mesenchymal stem cells were isolated and cultured as previously described (29). Briefly, GMSCs were liberated from the tissue by sequential digestion with 2mg/mL dispase II overnight and then 4mg/mL collagenase IV for 2 hr and then cultured in alpha-minimum essential medium (α-MEM) (Invitrogen, Carlsbad, CA, http://www.invitrogen.com) supplemented with 10% fetal bovine serum (FBS) (Clontech Lab, Inc., Mountain View, CA, http://www.clontech.com), 100 U/ml penicillin and 100 μg/ml streptomycin (Invitrogen). Cells from the fourth to sixth passages were used in experiments.

#### RASF

Human synovial tissue or fluid samples were collected from RA patients during synovectomy, arthroplasty or arthrocentesis under an approved IRB protocol. Tissue samples were digested with 4mg/mL collagenase IV for 1 hour and then mechanically dissociated with the GentleMACS (Miltenyi Biotec, Gaithersburg, MD). The cell suspension was diluted in DMEM and placed in culture for two days after which non-adherent cells were washed out and RASF were expanded. Experiments were performed with RASF between passages two and six.

### Exosome isolation and characterization

Exosomes were isolated from GMSC culture conditioned medium by ultracentrifugation as previously described (30). Briefly, GMSCs were cultured in exosome free medium for 2–3 days, the culture medium was then centrifuged at 300g for 10 min and the supernatant was subsequently centrifuged at 2000g for 10 min, then the supernatant was centrifuged at 10,000g for 30 min. The supernatant was then centrifuged at 100,000g for 70 min and the pellet was washed with PBS and then centrifuged again at 100,000g for 70 min. The exosomes utilized in these experiments were analyzed with a Nanosight NS300 and found to have an average size of 104.1 +/− 5.5 nm and an average concentration of 6.17×10^7^ +/− 5.81×10^6^ particles/mL.

#### Chimeric synovitis model:

Animals were group housed in Allentown NexGen IVC rack caging system (Allentown, NJ) on corncob bedding with a compressed cotton nestlet. Cages were autoclaved before use, irradiated feed (Envigo Teklad 7912) was provided ad libitum and reverse osmosis, chlorinated water was provided via automatic water valves. Cages were changed every two weeks in a biosafety cabinet and light was provided on a 12:12 cycle. Inhaled isoflurane 1–5% in 100% oxygen was used for anesthesia. Peri-operative analgesia included an NSAID, either 0.1ml meloxicam (0.5 mg/ml; 5mg/ml Metacam (Boehringer Ingelheim) diluted in 0.9% sterile saline) SQ or ibuprofen (100mg/5ml (Children’s Motrin) diluted 1:100 in drinking water) for at least 3 days post-operative, 1 drop of 0.25% bupivacaine (0.5% diluted 1:1 with sterile saline) along the skin incision before closure, and 0.05ml Buprenorphine SR-LAB (0.5 mg/ml, ZooPharm) SQ once at the time of surgery. Hair was removed from the dorsal cervical and scapular region using Nair (Church & Dwight Co., NJ) and the surgical site prepared using three alternating rounds of chlorhexidine and alcohol. A surgical plane of anesthesia was determined via unresponsiveness to a toe pinch and surgery was performed using aseptic technique.

Surgery of female severe combined immunodeficiency disease (SCID) mice was performed as previously described (18, 20, 21) with the following modifications. Human joint samples were obtained from osteoarthritis patients undergoing joint replacement surgery at The Ohio State University, Department of Orthopedics under an approved IRB protocol. The healthy articular cartilage/bone was cut away from the joint and then sliced into sections. Implants were assembled by inserting a 1mm^3^ – 2mm^3^ piece of cartilage/bone into a gelatin sponge (Pfizer, New York, NY) measuring approximately 3mm^3^ – 5mm^3^ which was wrapped in Bard surgical mesh (BD, Franklin Lakes, NJ) and sutured together. 5 × 10^5^ RASF in 50 μL sterile PBS or 50 μL of sterile PBS was then allowed to absorb into the sponge of the implants which were then stored in a humidified container at room temperature until implantation (< 4 hours). Each SCID mouse received two implants via a single transverse interscapular incision of approximately 5mm followed by blunt dissection; the implants containing RASF were subcutaneously implanted in the right flank while implants containing only PBS were implanted in the left flank. The incision was closed with 7mm or 9mm wound clips and the entire procedure lasted less than 30 minutes. Mice were monitored daily for at least 5 days post-operative and any animals that became moribund were removed and not included in the analysis. Dehisced surgical sites were reclosed once, or animals were removed from study. After 5–7 days mice were treated with either 5 × 10^5^ GMSCs or 3×10^6^ GMSC derived exosome particles via retro-orbital injection. Animals were euthanized via an overdose of carbon dioxide in accordance with the 2020 AVMA Euthanasia Guidelines. Implants were harvested at 60 days post-surgery and the cartilage was isolated from the other components of the implant. The cartilage was then fixed with 10% neutral buffered formalin and stored in 70% ethanol until being further processed for histology. 40X H&E stained digital slide images of cartilage sections were visualized and depth of penetration was measured by using Aperio ImageScope v12.1.0.5029.

### Histology and IHC

All samples were either processed at The Ohio State University, College of Veterinary Medicine, Histology/Immunohistochemistry Core Lab or at Histowiz (Brooklyn, NY) according to established protocols and the resulting slides were scanned at 40X magni cation.

### Flow Cytometry tracking of GMSC

GMSC were labeled with ViaFluor 405 (Biotium, Fremont, CA) and then injected intraperitoneally into female SCID mice that had received one implant containing cartilage and RASF 3 days earlier. Labeled GMSC were maintained in culture for the duration of the experiment. The implants were harvested at 4 days and the cartilage was isolated and digested in collagenase to release adherent cells. The cell suspension was analyzed on a BD FACSCelesta ow cytometer (BD, Franklin Lakes, NJ) and data were analyzed by using FlowJo v10.8.1 (Ashland, OR).

The cultured ViaFluor 405 labeled GMSC were acquired on the cytometer on days 1, 2, 3, and 4 to track the fluorescence intensity of the dye over the course of the experiment to identify a positive cell gating strategy for the cells harvested from the implants on Day 4.

### In vitro co-incubation of RASF with GMSC

RASF were labeled with ViaFluor 405 according to the manufacturers’ protocol and then 5,000 RASF were plated per well in a 24 well plate. GMSC were labeled with CFSE and then either 5,000 or 500 GMSC were added to the appropriate wells of the 24 well plate. The cells were allowed to co-incubate for 3 days and were trypsinized, resuspended in AnnexinV binding buffer, stained with AnnexinV-APC (Biolegend, San Diego, CA) and then analyzed on a BD FACSCelesta flow cytometer. For the cell crosstalk experiment, the RASF were separately labeled with ViaFluor 405 and the GMSC were labeled with ViaFluor 488. The cells were washed extensively and incubated in complete media in suspension for 1 hr. to ensure no residual dye reactivity was present. The cells were then added to a 24 well plate at the indicated ratios and cultured for two days. For the transwell incubation, the RASF were added to the bottom well and the GMSC were added to the top transwell chamber according to the manufacturer’s directions. The cells were cultured for 2 days and were then collected and analyzed on a BD FACSCelesta ow cytometer.

#### Statistics:

Data were analyzed with GraphPad Prism 9.4.0 (San Diego, CA) by using two-way ANOVA followed by Tukey’s multiple comparison testing. P-values < 0.05 were considered significant.

## Results

**Figure 1:**
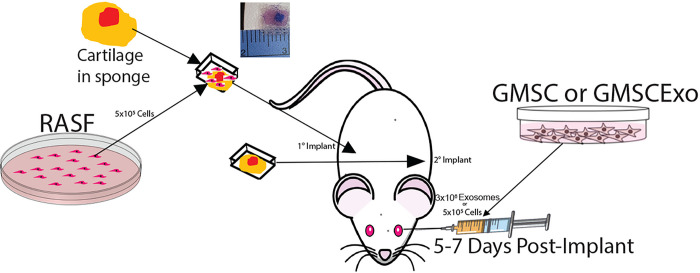
Schematic Overview of the Chimeric Mouse Model of Synovitis Cartilage-sponge-mesh implants with or without RASF were implanted subcutaneously in the right (1) or left (2) ank respectively. 5–7 days after the implantation surgery, the mice were retro-orbitally injected with GMSC or GMSCExo.

To evaluate the effects of both GMSC and GMSCExo on the ability of RASF to invade and degrade cartilage, a chimeric mouse model was used (Fig. 1). Implants were assembled from a small piece of human articular cartilage which was inserted into an absorbent gelatin sponge and then wrapped in surgical mesh. Two implants were made for each mouse, one was hydrated with 5×10^5^ RASF suspended in PBS and one was hydrated with PBS alone. The implant containing the RASF was implanted subcutaneously in the right flank (primary implant) of a SCID mouse and the cell-free implant with PBS alone was implanted in the left flank (secondary implant). The mice were allowed to recover from surgery and then were injected once with either GMSC or GMSCExo at 5–7 days post-implantation. The implants were removed after 60 days for evaluation.

**Figure 2:**
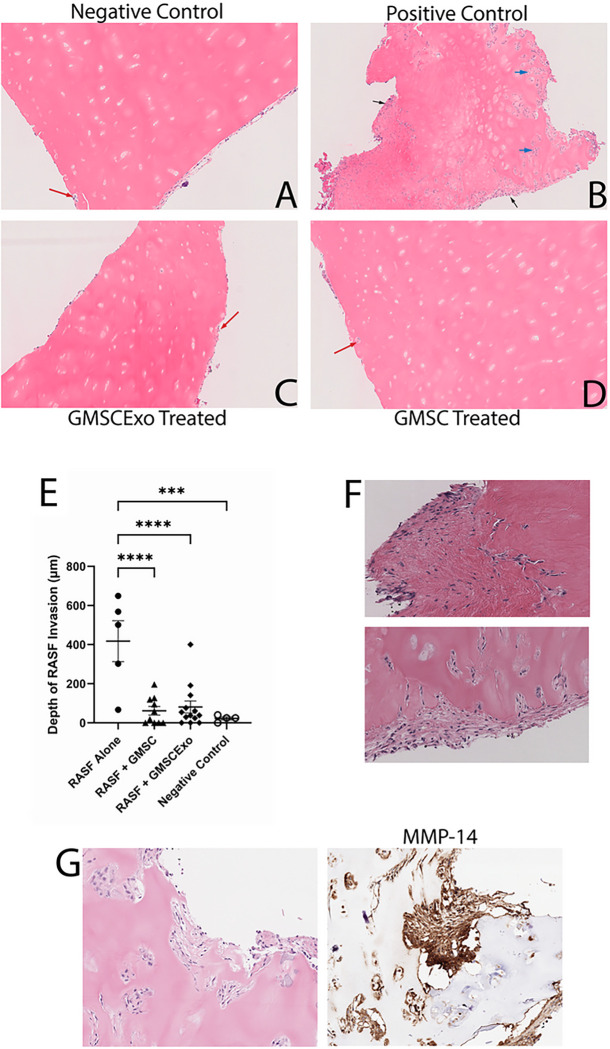
GMSC & GMSCExo Block RASF Cartilage Invasion in the Primary Implant (A) H&E stained negative control cartilage showing no RASF invasion, red arrow showing mouse cell occupation of a small pre-existing void in the cartilage. (B) H&E stained positive control cartilage showing deep RASF penetration throughout the tissue including perichondrocytic degradation, blue arrows, and large pannus formation, black arrows. (C) H&E stained RASF treated and GMSC exosome treated cartilage showing no RASF invasion with mouse cells occupying the surface of the tissue, red arrow. (D) H&E stained RASF treated and GMSC treated cartilage showing no RASF invasion with mouse cell occupying the surface of the tissue, red arrow. (E) Invasion depth demonstrates very deep penetration of the cartilage when treated with RASF alone. Treatment with either GMSC or GMSCExo blocks the invasion of the primary implant (mean±SE, n=5 for RASF Alone and Negative Control, n=10 for RASF + GMSC, n=13 for RASF + GMSCExo, ***p<0.0002; ****p<0.0001). (F) Close up images of the pannus structures. (G) Serial sections of RASF alone (positive control) treated cartilage showing expression of MMP-14.

Previous reports have demonstrated the invasive nature of RASF in this model of synovitis (18–21). Our studies recapitulated these invasive and destructive properties of RASF as seen in Fig. 2B. The black arrows indicate a rheumatoid pannus structure, as defined by an aggregate of cells forming on the surface of the cartilage with finger-like projections invading deeper into the cartilaginous tissue, which formed in 80% of the primary implants in the positive control mice. There was also extensive perichondrocytic invasion of the RASF as indicated by the blue arrows (Fig. 2B) that did not occur in the negative controls or the GMSC or GMSCExo treated mice. Figure 2F shows zoomed-in images of the pannus structures revealing the aggressive invasion and degradation of the cartilage by the RASF. This result did not occur when the implant contained negative control primary human dermal fibroblast cells, data not shown. In contrast to the positive control mice, none of the GMSC treated mice developed a pannus structure. In GMSCExo treated mice, 23% of the primary implants had pannus like structures. The red arrows point to areas of minimal cell infiltration into the cartilage in the negative controls and the GMSC and GMSCExo treated mice, but this phenomenon was very shallow and infrequent and appears as though mouse cells are occupying naturally occurring voids in the cartilage tissue (Fig. 2A, C & D). Treatment of the mice with either GMSC or GMSCExo inhibited the invasiveness of the RASF (Fig. 2C & D). Measurement of the depth of invasion revealed a highly significant inhibition of cartilage invasion by the RASF, similar to the negative control (Fig. 2E) that did not contain any RASF and therefore did not demonstrate any pannus structure or cartilage degradation.

The RASF in the synovium of rheumatoid arthritis patients have been shown to produce matrix metalloproteinases (MMPs) which facilitate the breakdown of the articular cartilage (19). MMP-14 is a type I transmembrane proteinase that is highly expressed on the cell surface of RASF in the joints of patients with RA and is considered a key enzyme that mediates cartilage invasion of RASF in RA (31). Therefore, we examined the expression of MMP-14 in the rheumatoid pannus structure and found that the majority of the invading cells highly express human MMP-14 (Fig. 2G).

**Figure 3:**
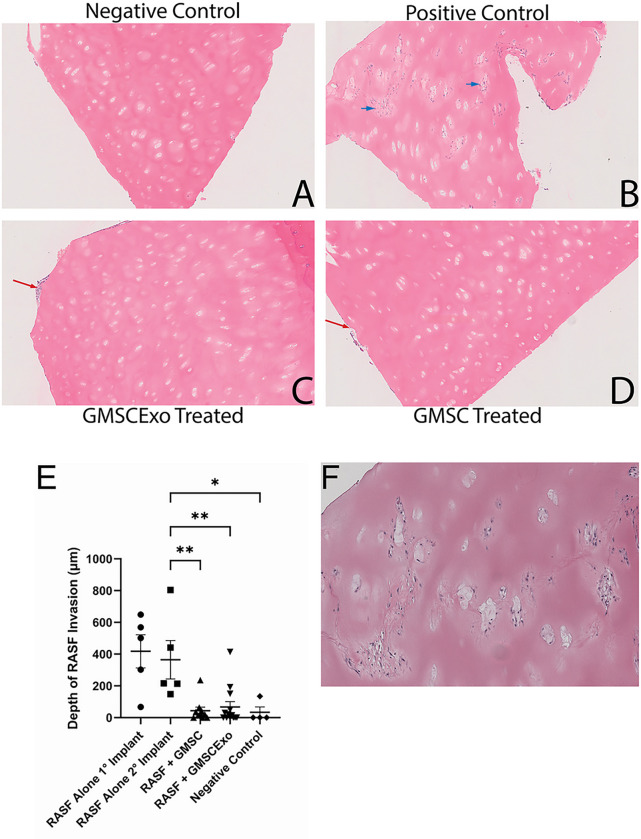
GMSC & GMSCExo Block Migration to and Invasion of the Secondary Implant (A) H&E stained negative control secondary cartilage showing no RASF invasion. (B) H&E stained positive control secondary cartilage showing deep RASF penetration throughout the tissue including perichondrocytic degradation, blue arrows. (C) H&E stained secondary cartilage section from a RASF treated, GMSC exosome injected mouse showing no RASF invasion with mouse cells occupying the surface of the tissue, red arrow. (D) H&E stained RASF treated and GMSC treated secondary cartilage showing no RASF invasion with mouse cells occupying the surface of the tissue, red arrow. (E) Invasion depth demonstrates very deep penetration of the secondary cartilage when treated with RASF alone. Treatment with either GMSC or GMSCExo blocks RASF invasion of the cartilage in the secondary implant (mean±SE, n=5 for RASF Alone 1°, 2° and Negative Control, n=10 for RASF + GMSC and RASF, n=13 for GMSCExo, *p<0.05; **p<0.01). (F) Close up image showing extensive perichondrocytic degradation in the RASF treated secondary location.

RASF have been shown to migrate out of the primary cartilage implant and into a secondary cartilage implant (21). This migratory capacity of the RASF is a possible mechanism by which the disease can start as monoarticular but quickly becomes polyarticular (12). The positive controls shown in Fig. 3B, and zoomed in image in 3F, demonstrate that not only is this result reproducible in our laboratory, but it is very robust. The RASF deeply invade the secondary cartilage implant that in most cases is approximately 2 cm away from the primary implant. In the positive control mice, the depth of penetration is no different from that of the primary implants (Fig. 3E – RASF Alone 1° Implant vs. RASF Alone 2° Implant). Interestingly, systemic treatment of the mice with either GMSC or GMSCExo inhibited the migration to and invasion of the RASF into the secondary cartilage implant (Fig. 3C & D). Measurement of the depth of invasion revealed a significant inhibition of RASF invasion into the secondary cartilage implant by both the GMSC and GMSCExo treatments (Fig. 3E).

**Figure 4:**
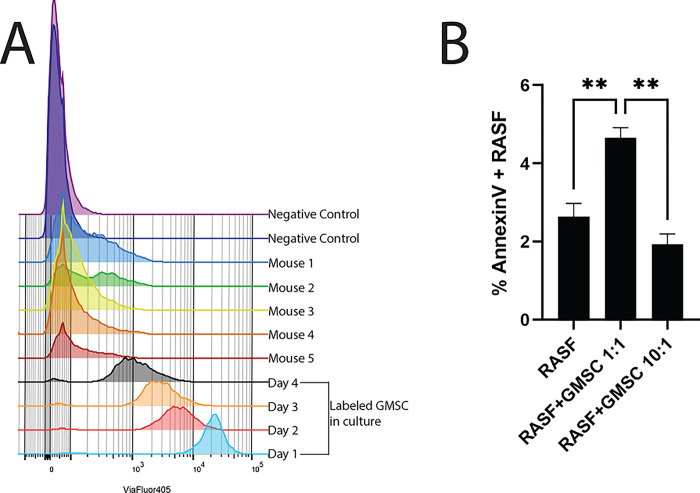
GMSCs Home to the Site of Implantation and Induce RASF PCD (A) Histogram overlay showing the ViaFluor 405 positive cells harvested from the implanted cartilage in the 5 mice injected with labeled GMSC. Negative controls show no positive cells while labeled GMSC maintained in culture show positive cells over the 4 day *in vivo* experiment. (B) In vitro experiment showing % RASF positive for annexinV. Co-incubation of RASF:GMSC at 1:1 ratio increased the occurrence of annexinV positive RASF, while a 10:1 ratio was no different from RASF alone (mean±SE, n=3).

**Figure 5:**
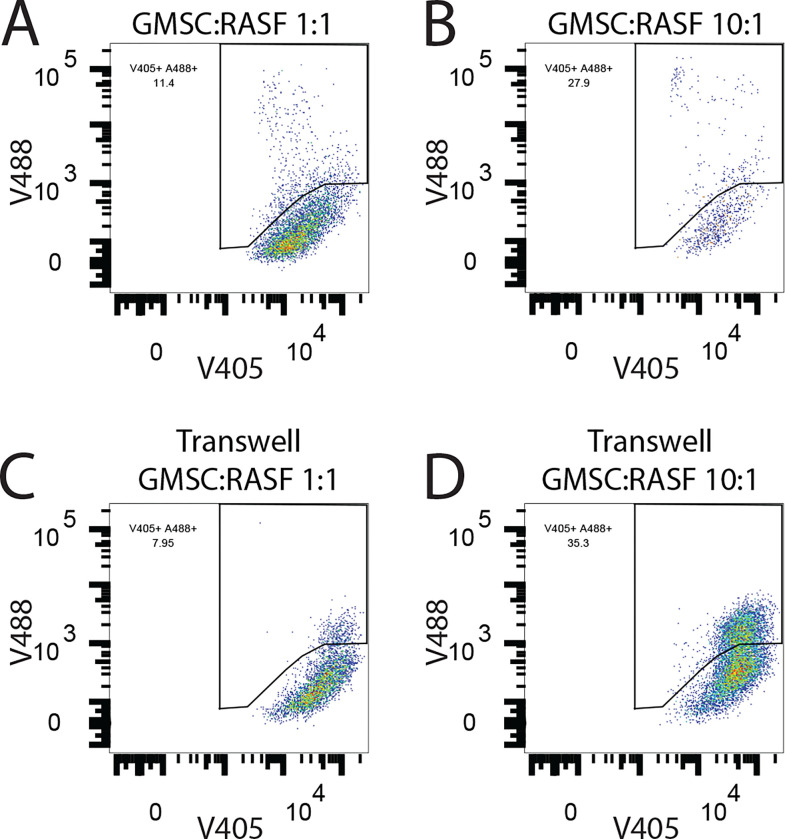
GMSCs Transfer Molecular Cargo *In Vitro* *In Vitro* co-incubation of GMSC with RASF shows transfer of the intracellular label ViaFluor488 from the GMSC to the RASF when incubated together in wells with no separation, A & B, or in transwells where cells are separated, C & D. The label is transferred in a ratio dependent manner in which increasing the GMSC:RASF ratio increases the number of RASF that become positive for the GMSC label, B & D. Data are representative of two experiments performed in triplicate.

We hypothesized that a possible mechanism of action by which the GMSC can inhibit the invasiveness and migratory action of the RASF was by homing to the primary site and directly interacting with the RASF. To test this hypothesis, SCID mice that received a single implant containing RASF were retro-orbitally injected with ViaFluor 405 labeled GMSCs. After 4 days post-injection of the GMSCs, the implants were harvested, digested with collagenase to liberate the cells and the resulting cell suspension was analyzed by flow cytometry. The original excess labeled cells were maintained in culture for the course of the experiment and analyzed in parallel to create a gating strategy to identify positive cells in the *in vivo* experimental samples. In addition, two negative control mice were injected with unlabeled GMSCs to establish a baseline for negative cells as well as to rule out any possible autofluorescent false positive cells. Five mice were tested in this scenario, and all five had ViaFluor 405 positive GMSCs in the implant at time of harvest (Fig. 4A). Since we were able to show that the GMSC can home to the primary site where the RASF were exerting their deleterious effects, we next wanted to determine how these stem cells could potentially inhibit the effects of the RASF. One possible explanation was that the GMSC were inducing programmed cell death (PCD) of the RASF. This would block both the cartilage invasion and the migration of the RASF to secondary sites. To test this hypothesis, we first labeled RASF with ViaFluor 405 *in vitro*, and co-incubated them at a 1:1 or 10:1 ratio with GMSC in cell culture for 3 days and then measured the levels of AnnexinV positivity in the fluorescently labeled RASF by flow cytometry. This co-incubation with GMSC led to a 76% increase in apoptotic cells as compared to RASF maintained in culture alone, whereas a lower ratio of 10:1 RASF:GMSC had no effect on the rate of RASF PCD (Fig. 4B). Next, we wanted to determine if the GMSC were possibly exerting this PCD effect on the RASF via cell communication through exosomes since we had already shown that the exosomes derived from the GMSC were also effective at inhibiting the deleterious effects of the RASF in the in vivo model. To do this, we separately labeled the RASF with ViaFluor 405 and the GMSC with ViaFluor 488 and then co-incubated them in culture for 2 days. This was done in a direct cell-cell contact culture as well as in transwells where the cells were not in direct contact. Our hypothesis was that if the GMSC were communicating with the RASF through exosomes, then the fluorescent label inside the GMSCExo would be transferred into the RASF and would be detectable via flow cytometry. Indeed, this was the case as is shown in Fig. 5A where the ViaFluor 488 signal was detectable in 11.8% ± 0.3% of the RASF in a culture with a ratio of GMSC:RASF at 1:1 at 48 hrs. Increasing the ratio of GMSC:RASF to 10:1 resulted in 30.3% ± 1.2% of the RASF becoming positive (Fig. 5B). Separating the cells in culture by the use of transwells resulted in 6.7% ± 2.0% of the RASF containing the GMSC label when incubated at a ratio of 1:1 (Fig. 5C), while in the 10:1 GMSC:RASF ratio condition, 37% ± 1.7% of the RASF contained the GMSC label (Fig. 5D).

## Discussion

RASF are one of the major cell types involved in the progressive joint destruction associated with RA and have been shown to effectively spread the disease from one joint to another (21). This progressive pathology can lead to debilitating results for patients who do not respond to current therapies and thus RASF are a major focus of research as potential therapeutic targets because of the major role that they play in RA pathobiology (9, 17, 32). Gingival derived mesenchymal stem cells have emerged as potential cellular therapies for various autoimmune diseases because of their now well-characterized immunomodulatory properties (22, 24, 27, 28). In addition, exosomes derived from these cells have been shown to possess many of the immunomodulatory properties of their parent cells and are therefore potentially better therapeutics as they do not possess the negative characteristics of cell-based therapies (33). Here, in a chimeric model of synovitis that occurs during the disease progression of rheumatoid arthritis, we have demonstrated that GMSC as well as exosomes derived from GMSC are promising therapeutics for the treatment of this debilitating disease.

The modulatory effects of the GMSC on RASF could be multiple including the induction of cell death, reduction in proliferation, modulating the activated invasive phenotype via paracrine secretion of cytokines and chemokines, transfer of molecular cargo via exosomes, etc. The mechanism by which this occurs has not been completely elucidated, however we present evidence that demonstrates several of these possibilities. The robust inhibition of both the invasion of RASF into the primary implant and the inhibition of the migration and invasion of the secondary implant by GMSCExo suggests that one of these mechanisms is the transfer of molecular cargo via exosomes. We have also established that GMSC will home to the site of cartilage implantation where the RASF reside and directly transfer their molecular cargo into the RASF. In addition, we show that when GMSC are co-incubated with RASF *in vitro*, there is a significant increase in the rate of RASF PCD suggesting that this induction of RASF cell death is an additional mechanism of action whereby the GMSC are exerting their inhibitory effects *in vivo*.

The GMSCExo treatment was less effective than GMSC in inhibiting the formation of the pannus structure in the primary implant. The explanation could be multifactorial, however the live GMSCs will continually secrete exosomes once they home to the implant, while, in these experiments, a single dose of exosomes was used. There is a possibility that a combination of the GMSC mechanisms of action are needed to be fully effective at blocking the formation of the pannus as GMSCs are fully functional cells able to secrete exosomes, induce RASF PCD and also potentially modulate RASF invasiveness via paracrine secretion of immunomodulatory factors. Interestingly, the depth of RASF invasion into the cartilage on average was not statistically significant between GMSC and GMSCExo for either the primary implant or the secondary implant which demonstrates that the potency of the exosome treatment is very close to the GMSC treatment.

## Conclusions

Our results indicate that not only the GMSC themselves, but also GMSCExo are able to block the pathological effects of RASF in this *in vivo* chimeric mouse model of the disease. We demonstrate that a single dose of either GMSC or GMSCExo can significantly inhibit the direct deleterious effects of RASF on cartilage in this model. In addition, these treatments were also able to block the invasive migration of the RASF, suggesting that they could potentially be used to treat patients that present with RA in the early phases of the disease process with a single or few joints affected and inhibit spreading to other joints, essentially blocking progression of the disease in its tracks. Because the gingival tissue is harvested with little difficulty, relatively small amounts of tissue are required to expand the cells, the fairly simple *in vitro* expansion process, and the increasing technological advances in the production of therapeutic exosomes, we believe that both GMSC and GMSCExo are excellent candidates for the treatment for RA.

## Data Availability

The datasets used and/or analyzed during the current study are available from the corresponding author on reasonable request.

## Abbreviations

RA: Rheumatoid arthritis
RASF: activated synovial fibroblasts
GMSC: gingival-derived mesenchymal stem cells
GMSCExo: exosomes derived from GMSCs
IRB: Institutional Review Board
SCID: severe combined immunodeficiency disease
MMPs: metalloproteinases
PCD: programmed cell death

## References

[1] Smolen JS, Aletaha D, Barton A, Burmester GR, Emery P, Firestein GS, Rheumatoid arthritis. Nat Rev Dis Primers. 2018;4:18001. 29417936 10.1038/nrdp.2018.1

[2] Aletaha D, Smolen JS. Diagnosis and Management of Rheumatoid Arthritis: A Review. JAMA. 2018;320(13):1360–72. 30285183 10.1001/jama.2018.13103

[3] Sepriano A, Kerschbaumer A, Bergstra SA, Smolen JS, Van der Heijde D, Caporali R, Safety of synthetic and biological DMARDs: a systematic literature review informing the 2022 update of the EULAR recommendations for the management of rheumatoid arthritis. Ann Rheum Dis. 2023;82(1):107–18. 36376026 10.1136/ard-2022-223357

[4] Tamas MM, Filippucci E, Becciolini A, Gutierrez M, Di Geso L, Bonfiglioli K, Bone erosions in rheumatoid arthritis: ultrasound findings in the early stage of the disease. Rheumatology. 2014;53(6):1100–7. 24501246 10.1093/rheumatology/ket484

[5] Shah P, Siddique A, Thakkar A, Gharat S, Godad A, Kale P, An update on novel therapeutic intervention in Rheumatoid arthritis. Int Immunopharmacol. 2022;109:108794. 35504203 10.1016/j.intimp.2022.108794

[6] Mankia K, Di Matteo A, Emery P. Prevention and cure: The major unmet needs in the management of rheumatoid arthritis. J Autoimmun. 2020;110. 10.1016/j.jaut.2019.102399 31899021

[7] Wu D, Luo YH, Li T, Zhao XY, Lv T, Fang G, Systemic complications of rheumatoid arthritis: Focus on pathogenesis and treatment. Front Immunol. 2022;13. 10.3389/fimmu.2022.1051082 PMC9817137 36618407

[8] Tsaltskan V, Firestein GS. Targeting fibroblast-like synoviocytes in rheumatoid arthritis. Curr Opin Pharmacol. 2022;67. 10.1016/j.coph.2022.102304 PMC9942784 36228471

[9] Bartok B, Firestein GS. Fibroblast-like synoviocytes: key effector cells in rheumatoid arthritis. Immunol Rev. 2010;233(1):233–55. 20193003 10.1111/j.0105-2896.2009.00859.x PMC2913689

[10] Matsuda K, Shiba N, Hiraoka K. New Insights into the Role of Synovial Fibroblasts Leading to Joint Destruction in Rheumatoid Arthritis. Int J Mol Sci. 2023;24(6). 10.3390/ijms24065173 PMC10049180 36982247

[11] Aghakhani S, Soliman S, Niarakis A. Metabolic reprogramming in Rheumatoid Arthritis Synovial Fibroblasts: A hybrid modeling approach. Plos Comput Biol. 2022;18(12). 10.1371/journal.pcbi.1010408 PMC9779668 36508473

[12] Lefevre S, Meier FMP, Neumann E, Muller-Ladner U. Role of Synovial Fibroblasts in Rheumatoid Arthritis. Curr Pharm Design. 2015;21(2):130–41. 10.2174/1381612820666140825122036 25163744

[13] Gao Y, Cai WW, Zhou Y, Li YH, Cheng JW, Wei F. Immunosenescence of T cells: a key player in rheumatoid arthritis. In amm Res. 2022;71(12):1449–62. 10.1007/s00011-022-01649-0 36280621

[14] Masoumi M, Alesaeidi S, Khorramdelazad H, Behzadi M, Baharlou R, Alizadeh-Fanalou S, Role of T Cells in the Pathogenesis of Rheumatoid Arthritis: Focus on Immunometabolism Dysfunctions. Inflammation. 2023;46(1):88–102. 36215002 10.1007/s10753-022-01751-9

[15] Suwa Y, Nagafuchi Y, Yamada S, Fujio K. The role of dendritic cells and their immunometabolism in rheumatoid arthritis. Front Immunol. 2023;14. 10.3389/fimmu.2023.1161148 PMC10213288 37251399

[16] Tu JJ, Huang W, Zhang WW, Mei JW, Zhu C. Two Main Cellular Components in Rheumatoid Arthritis: Communication Between T Cells and Fibroblast-Like Synoviocytes in the Joint Synovium. Front Immunol. 2022;13. 10.3389/fimmu.2022.922111 PMC9284267 35844494

[17] Muller-Ladner U, Ospelt C, Gay S, Distler O, Pap T. Cells of the synovium in rheumatoid arthritis. Synovial fibroblasts. Arthritis Res Ther. 2007;9(6):223. 18177509 10.1186/ar2337 PMC2246247

[18] Judex M, Neumann E, Fleck M, Pap T, Mountz JD, Gay RE, “Inverse wrap”: an improved implantation technique for virus-transduced synovial fibroblasts in the SCID mouse model for rheumatoid arthritis. Mod Rheumatol. 2001;11(2):145–50. 24383692 10.3109/s101650170027

[19] Miller MC, Manning HB, Jain A, Troeberg L, Dudhia J, Essex D, Membrane Type 1 Matrix Metalloproteinase Is a Crucial Promoter of Synovial Invasion in Human Rheumatoid Arthritis. Arthritis Rheum-Us. 2009;60(3):686–97. 10.1002/art.24331 PMC2819053 19248098

[20] Muller-Ladner U, Kriegsmann J, Franklin BN, Matsumoto S, Geiler T, Gay RE, Synovial fibroblasts of patients with rheumatoid arthritis attach to and invade normal human cartilage when engrafted into SCID mice. Am J Pathol. 1996;149(5):1607–15. 8909250 PMC1865262

[21] Lefevre S, Knedla A, Tennie C, Kampmann A, Wunrau C, Dinser R, Synovial fibroblasts spread rheumatoid arthritis to unaffected joints. Nat Med. 2009;15(12):1414–20. 19898488 10.1038/nm.2050 PMC3678354

[22] Gan L, Liu Y, Cui DX, Pan Y, Zheng LW, Wan M. Dental Tissue-Derived Human Mesenchymal Stem Cells and Their Potential in Therapeutic Application. Stem Cells Int. 2020;2020. 10.1155/2020/8864572 PMC7482010 32952572

[23] Huang F, Liu ZM, Zheng SG. Updates on GMSCs Treatment for Autoimmune Diseases. Curr Stem Cell Res T. 2018;13(5):345–9. 10.2174/1574888X13666180220141114 29468980

[24] Tomar GB, Srivastava RK, Gupta N, Barhanpurkar AP, Pote ST, Jhaveri HM, Human gingivaderived mesenchymal stem cells are superior to bone marrow-derived mesenchymal stem cells for cell therapy in regenerative medicine. Biochem Bioph Res Co. 2010;393(3):377–83. 10.1016/j.bbrc.2010.01.126 20138833

[25] Yin F, Battiwalla M, Ito S, Feng XM, Chinian F, Melenhorst JJ, Bone Marrow Mesenchymal Stromal Cells to Treat Tissue Damage in Allogeneic Stem Cell Transplant Recipients: Correlation of Biological Markers with Clinical Responses. Stem Cells. 2014;32(5):1278–88. 24452962 10.1002/stem.1638 PMC3991733

[26] Huang F, Chen MG, Chen WQ, Gu J, Yuan J, Xue YQ, Human Gingiva-Derived Mesenchymal Stem Cells Inhibit Xeno-Graft-versus-Host Disease via CD39-CD73-Adenosine and IDO Signals. Front Immunol. 2017;8. 28210258 10.3389/fimmu.2017.00068 PMC5288353

[27] Tian XH, Wei WM, Cao Y, Ao TR, Huang F, Javed R, Gingival mesenchymal stem cell-derived exosomes are immunosuppressive in preventing collagen-induced arthritis. J Cell Mol Med. 2022;26(3):693–708. 34953015 10.1111/jcmm.17086 PMC8817124

[28] Zheng SG, Luo Y, Wang JL. Human gingival tissue-derived MSC suppress osteoclastogenesis and bone erosion via CD39-adnosine signal pathway in autoimmune arthritis. J Immunol. 2019;202(1). 10.1016/j.ebiom.2019.04.058 PMC6558261 31076346

[29] Zhang QZ, Shi SH, Liu Y, Uyanne J, Shi YF, Shi ST, Mesenchymal Stem Cells Derived from Human Gingiva Are Capable of Immunomodulatory Functions and Ameliorate Inflammation-Related Tissue Destruction in Experimental Colitis. J Immunol. 2009;183(12):7787–98. 19923445 10.4049/jimmunol.0902318 PMC2881945

[30] Thery C, Amigorena S, Raposo G, Clayton A. Isolation and characterization of exosomes from cell culture supernatants and biological fluids. Curr Protoc Cell Biol. 2006;Chap. 3:Unit 3 22. 10.1002/0471143030.cb0322s30 18228490

[31] Sabeh F, Fox D, Weiss SJ. Membrane-Type I Matrix Metalloproteinase-Dependent Regulation of Rheumatoid Arthritis Synoviocyte Function. J Immunol. 2010;184(11):6396–406. 20483788 10.4049/jimmunol.0904068

[32] Pap T, Muller-Ladner U, Gay RE, Gay S. Fibroblast biology. Role of synovial fibroblasts in the pathogenesis of rheumatoid arthritis. Arthritis Res. 2000;2(5):361–7. 11094449 10.1186/ar113 PMC130137

[33] Liu H, Li RC, Liu T, Yang LY, Yin G, Xie QB. Immunomodulatory Effects of Mesenchymal Stem Cells and Mesenchymal Stem Cell-Derived Extracellular Vesicles in Rheumatoid Arthritis. Front Immunol. 2020;11. 32973792 10.3389/fimmu.2020.01912 PMC7468450

